# Hemodynamic Changes Before and After Endovascular Treatment of Type B Aortic Dissection by 4D Flow MRI

**DOI:** 10.3389/fcvm.2022.873144

**Published:** 2022-05-25

**Authors:** Benoit Cosset, Loic Boussel, Eduardo Davila Serrano, Antoine Millon, Philippe Douek, Fadi Farhat, Monica Sigovan

**Affiliations:** ^1^University Lyon 1, INSA Lyon, UCBL 1, CNRS, Inserm, CREATIS UMR 5220, Lyon, France; ^2^Department of Cardio-Vascular Surgery, Hospices Civils de Lyon, Lyon, France; ^3^Department of Radiology, Hospices Civils de Lyon, Lyon, France; ^4^Department of Vascular Surgery, Hospices Civils de Lyon, Lyon, France

**Keywords:** 4D Phase-Contrast MRI, type B aortic dissection, thoracic endovascular aortic repair (TEVAR), hemodynamic evaluation, helical flow, parametric hemodynamic maps

## Abstract

**Objective:**

The standard treatment for complicated Stanford type B aortic dissection (TBAD) is thoracic endovascular aortic repair (TEVAR). Functional parameters, specifically blood flow, are not measured in the clinical assessment of TEVAR, yet they are of outmost importance in patient outcome. Consequently, we investigated the impact of TEVAR on the flows in the aorta and its branches in TBAD using 4D Phase-Contrast Magnetic Resonance Imaging (4D Flow MRI).

**Methods:**

Seven patients with TBAD scheduled for TEVAR underwent pre and post-operative 4D Flow MRI. An experienced reader assessed the presence of helical flow in the false lumen (FL) using streamlines and measured net flow at specific locations. In addition, forward and reverse flows, stasis, helicity, and absolute helicity were computed automatically along the aorta centerline. Average values were then computed in the segmented vessels. Impact of TEVAR on these parameters was assessed with a Wilcoxon signed rank test. Impact of the metallic stent on the velocity quantification was assessed using intra-class correlation coefficient (ICC) between velocities measured intra-stent and in adjacent stent-free regions.

**Results:**

FL helical flow was observed proximally in 6 cases and distally in 2 cases pre-operatively. Helical flow disappeared post-TEVAR proximally, but developed distally for 2 patients. Intra-stent measures were similar to stent-free with a median difference of 0.1 L/min and an ICC equal to 0.967 (*p* < 0.01). Forward flow increased from 59.9 to 81.6% in the TL and significantly decreased in the FL from 15.9 to 3.3%. Similarly, reverse flow increased in the TL from 4.36 to 10.8% and decreased in the FL from 10.3 to 4.6%. No significant changes were observed in net flow for aortic branches (*p* > 0.05). A significant increase in FL stasis was observed (*p* = 0.04).

**Discussion:**

TEVAR significantly increased forward flow in the TL and significantly decreased both forward and reverse flows in the FL. Interestingly, reverse flow in the TL increased post-TEVAR, which could be due to increased rigidity of the wall, due to the metallic stent. User independent helicity quantification enabled detection of elevated helicity at the level of secondary entry tears which had been missed by streamline visualization.

## Purpose

Aortic dissection (AD) is a serious pathology affecting one in 30 000 people per year in western countries (1). Aortic dissection involves the formation of a tear in the intima of the aortic wall, which enables blood to penetrate the wall and separates the media in two parts, creating a new channel, called false lumen. The false lumen will then extend all along the aorta. According to the Stanford classification, type A dissections concern the ascending aorta, while type B dissections concern only the descending aorta. Clinical management of all type A dissections (TAAD) is emergency surgical intervention, while for type B dissections (TBAD) management involves imaging follow-up, medical therapy, and endovascular treatment in complicated cases. Despite standardized management, short- and long-term survival for aortic dissection patients is low. Thus, in-hospital and 10 years mortality are respectively around 21.7 and 52.2% for TAAD and 14 and 59–66% for TBAD (2–4). Moreover, the rate of complications in high in aortic dissections. Short term complications include persistent malperfusion, retrograde dissection, stroke, organ failure, paraplegia, and type I endoleaks (5). Long term complications are aneurismal evolution and re-intervention, the high rate of which are around 43% 8 years after TAAD (6), 33% 10 years for TBAD following intervention and 41% 10 years for medically treated TBAD (4).

Imaging follow-up is performed with either CT angiography or MR angiography, both of which only provide morphological markers for characterizing what is essentially a hemodynamic pathology. The main complication of TBAD is aneurysmal progression, which may lead to aortic rupture. The diameter of the false lumen and the amount of false lumen thrombosis are used to assess the risk of rupture and thus select patients for intervention. Morphological imaging parameters, used in current clinical practice, are related to a presumed change in flow in the false lumen, for example thrombus formation is related to low flow, and an increase in diameter to high flow. However, hemodynamic parameters, such as velocity, flow rate, and flow pattern in the false lumen and through the main entry tear, have a role in aneurysm formation, as demonstrated by imaging, biomechanical, and modeling studies.

4D Phase Contrast MRI (4D PCMRI, or 4D Flow), albeit not yet part of clinical routine, provides direct measurements of velocity, enabling visualization and accurate quantification of blood flow characteristics in patients with aortic dissection (7, 8). Importantly, this functional information has the potential to improve AD management. Flow features obtained with 4D Flow have been related to the risk of developing complications (9, 10). Clough et al. (9) demonstrated that helicity, particularly the amount of helicity in the false lumen, is correlated with the rate of aortic expansion. The regurgitation flow through the primary entry tear appears as another interesting aneurismal risk factor, demonstrated recently by Burris et al. (10) in 18 chronic dissection cases.

When complications are identified during surveillance, thoracic endovascular aortic repair (TEVAR) is generally performed (11, 12). The objective of TEVAR is to close the main intimal tear that is expected to result in an increase of the thoracic true lumen diameter, a decrease of the thoracic false lumen diameter, and improved perfusion of the aortic branches (12). However, the impact of TEVAR on the different flow features mentioned above on an individual patient basis is not clear. Consequently, our goal was to use 4D Flow MRI to investigate how the flow features in aortic dissections change as a result of TEVAR in TBAD patients.

## Methods

### Study Population

Patients with an aortic dissection requiring endovascular treatment or hybrid surgery according to surgical guidelines (5, 13, 14) were prospectively included between June 2018 and May 2020. Patients were identified during their hospitalization at our institution or during a consultation with a cardio-vascular surgeon. They were included after multidisciplinary staff screening. Patients younger than 18 years old, patients requiring an emergency intervention, patients with contra-indication to endovascular treatment, and/or contra-indication to MRI (claustrophobia, ferromagnetic materials: pacemaker, prosthetic heart valve, implantable cardioverter defibrillators, vascular clip), pregnancy or breastfeeding, dementia were excluded. Written informed consent was obtained from all included patients. Institutional review board approval was obtained (ID-RCB: 2018-A01742-53).

### Study Design

After inclusion in the study, patients underwent a clinical examination and were scheduled for endovascular treatment. All patients underwent a baseline MRI scan prior to the procedure and a follow-up MRI within 6 month after the intervention.

We collected prospectively clinical data (personal and familial history, vascular clinical exam, blood pressure, pain assessment at thoracic, abdominal and lower body level), biological data (renal function, arterial lactates) and radiological data (DeBakey and Stanford classification, presence of haemothorax, signs of aortic rupture, maximal thoracic aortic diameter, signs of malperfusion).

### Image Acquisition

MRI was performed on an Ingenia 3T system (Philips, Best, The Netherlands). The protocol included dynamic gadolinium-enhanced angiography (4DTRAK) (6) and 4D Flow (14). 4D TRAK was performed using a 3D spoiled gradient echo sequence with the following imaging parameters: TR = 3.8 ms, TE = 1.1 ms, spatial resolution = 0.75 x 0.75 x 2 mm, slice overlap 1 mm, 30 frames, temporal resolution = 1.3 s, flip angle 25°. Acquisition was performed 5 s after intra-venous administration of 2 cc/kg of a gadolinium contrast agent (DOTAREM, Guerbet, France). Patients were instructed to hold their breath during the early dynamic phases and breathe out slowly during the last phases. 4D Flow was performed using a retrospective ECG gated and respiratory navigated spoiled gradient echo sequence covering the aorta from the supra-aortic vessels to below the renal arteries in a sagittal oblique orientation. Acquisition parameters were as follows: TR = 4–4.6 ms, TE = 2.2–2.7 ms, flip angle = 8°, spatial resolution = 2.5–2.8 isotropic mm^3^, velocity sensitivity (VENC) = 80–200 cm/s. Twenty cardiac phases were reconstructed for each patient.

ECG-gated contrast enhanced CT angiography was available in all the patients for both pre- and post-intervention.

### Data Analysis

Qualitative analysis of the 4D Flow MRI data was performed with dedicated commercial software, Caas MR Solution (Pie medical imaging, Maastricht, Netherlands). Background phase and velocity aliasing corrections were automatically performed by the software. Time averaged speed was used to visualize and semi-automatically segment the aorta. A descriptive analysis of the flow patterns was performed using streamline visualization. Helical flow, defined as the longitudinal evolution of flow rotating around an axis parallel to the main axis of the vessel, was recorded as present or absent in both pre- and post-operative exams in the true lumen, in the false lumen from the isthmic aorta to the infra-renal aorta. Subsequently, a quantitative analysis of helicity was performed as part of the quantitative analysis described in the following.

Quantitative analysis was performed using code developed in Matlab (The MathWorks, Natick, MA, USA). The analysis was based on recent methodology proposed by Jarvis and colleagues (15). Briefly, manual segmentation of the aorta was performed on computed phase-contrast MR Angiograms. Affine registration of the time averaged 4DTRAK volume was used to guide the segmentation when necessary. On post-TEVAR images, the stent related localized phase noise was identified by computing the FH velocity image spatial gradient and then summing it in the temporal dimension. Pixels presenting with high image gradients (>8 kHz) were then set to zero in the mask. The true and the false lumen were segmented separately. Cross-sections were placed automatically along the vessel centerline spaced 1 mm apart. Centerline was computed using an iterative thinning algorithm applied to the mask of the aorta (TL and FL combined). For each pixel of the segmented mask and across the cardiac cycle, the direction of flow was obtained with respect to the centerline either forward (ascending to descending) or reverse (descending to ascending). The net, forward, and reverse flows were measured per cross-section. Forward flow (*FF*) and reverse flow (*RF*) maps were then computed as the sum over the cardiac cycle. Flow stasis was defined as percentage time over the cardiac cycle with velocity < 0.1 m/s. Quantification of helicity was performed using the same segmented mask. Helicity (*H*) is defined as the integrated scalar product of the velocity vector and the vorticity vector (the curl of the velocity field) and it quantifies the rotation about an axis parallel to the main direction of flow: *H* = *v*(*r, t*)∙ω(*r, t*). The sign of the helicity value indicates the direction of rotation. Helicity and absolute helicity values were obtained along the aortic centerline only in the false lumen. 2D average intensity projection images were obtained for each parameter map (*FF, RF, Stasis, H, absH*) to facilitate visualization.

The analysis described above was performed in the aorta from the ascending part (5 cm above the sinus) to the infra-renal part. The aortic branches were not included in the segmentation. Thus, a local quantitative analysis was performed by manually placing 2D cross-sectional planes perpendicular to the vessel centerline and computing net flows using the Caas MR Solutions software. Finally, flow values were normalized to the net flow of the ascending aorta to allow direct comparisons between patients and time points. Relative changes in the net flow of the true lumen, the false lumen, and the aortic branches were obtained as the difference between post- and pre-TEVAR normalized to the pre-TEVAR values.

### Statistical Analysis

The impact of the endovascular treatment on the flows in the true lumen (TL), false lumen (FL), visceral branches, renal arteries, was assessed using a non-parametric test, Wilcoxon signed rank test, since normality of the data was not assumed.

In order to evaluate the impact of the metallic stent on the velocity quantification, intra-class correlation coefficient was computed between velocity measured intra-stent and velocity measured in adjacent stent-free regions.

## Results

### Population

Seven patients were included between June 2018 And May 2020. The first MRI exam was performed 29 (+/-56) days before the treatment, the second MRI exam was performed 30 (+/- 36) days after the treatment. Mean age was 58 +/- 19 years, 6 patients (85.7%) were males, all patients had a history of hypertension, 1 patient (14.3%) had a history of smoking, 3 (42.8%) patients had dyslipidemia history, none had family history of aortic dissection, 1 patient (14.3%) had a Marfan syndrome. Indication of surgical treatment (TEVAR only or TEVAR + stabilize technique) was aneurismal evolution for 2 patients (28.5%), malperfusion syndrome for 3 patients (42.8%), haemothorax for 1 patient (14.3%), persistent pain for 1 patient (14.3%). Description of patient population is presented in Table 1.

**Table 1:** Baseline clinical and procedural characteristics of patients with type B aortic dissection and indication of endovascular treatment.

	**No. (%) of Patients (*N* = 7)**
**Population characteristics**	
Age	58.4 (+/-18.9)
Male	6 (85.7%)
Hypertension	7 (100%)
Smoke	1 (14.3%)
Dyslipidemia	3 (42.8)
Marfan syndrome	1 (14.3%)
**Indication**	
Aneurysmal evolution	2 (28.5%)
Malperfusion syndrome	3 (42.8%)
Hemothorax	1 (14.3%)
Persistent pain	1 (14.3%)
**Time after dissection onset**	
Acute (<14 days)	2 (28.5%)
Sub-acute (14–90 days)	0
Chronic (>90 days)	5 (71.5%)
**Surgical procedure characteristics**	
TEVAR only	4 (57.2%)
TEVAR + stabilize technique	3 (42.8%)

### Surgery

Two (28.5%) patients were treated in the acute phase (<14 days) and five (71.5%) in the chronic phase (>90 days). The same surgeon treated all patients as follows: the endoprosthesis was implanted from the aortic isthmus (distally to the left sub-clavicular artery) to the diaphragmatic level of the aorta (proximally to the truncus coeliacus) with the objective of closing the primary entry tear. For three patients (42.8%), a bare stent was used in addition to the TEVAR, in distality of the thoracic aorta, this is known as the stabilize technique as described by Hofferbeth et al. (16).

### Flow Analysis

**Figure 1:**
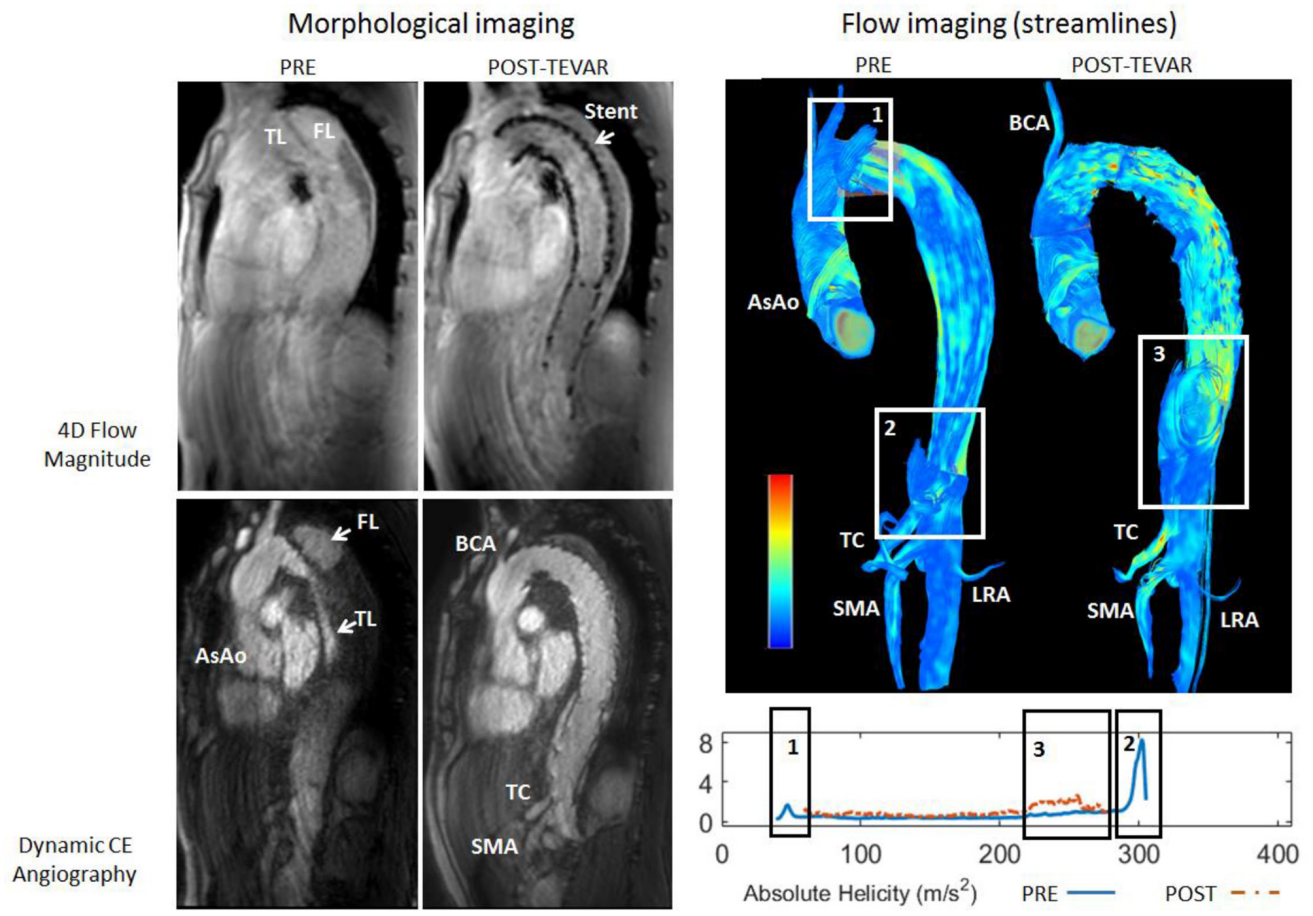
Top: Streamline visualization of the pre-operative and post-operative flow patterns in the aorta of Patient 4. Pre-operatively, presence of helical flow can be observed in the false lumen resulting from the primary entry tear (rectangle 1) and at the ostium of the truncus caeliacus (TC, rectangle 2). Post-operatively, presence of helical flow can be observed in the false lumen downstream of the endo-prosthesis at the diaphragmatic level (rectangle 3). Bottom: Plots of absolute helicity along the false lumen, in agreement with the qualitative observations.

**Figure 2:**
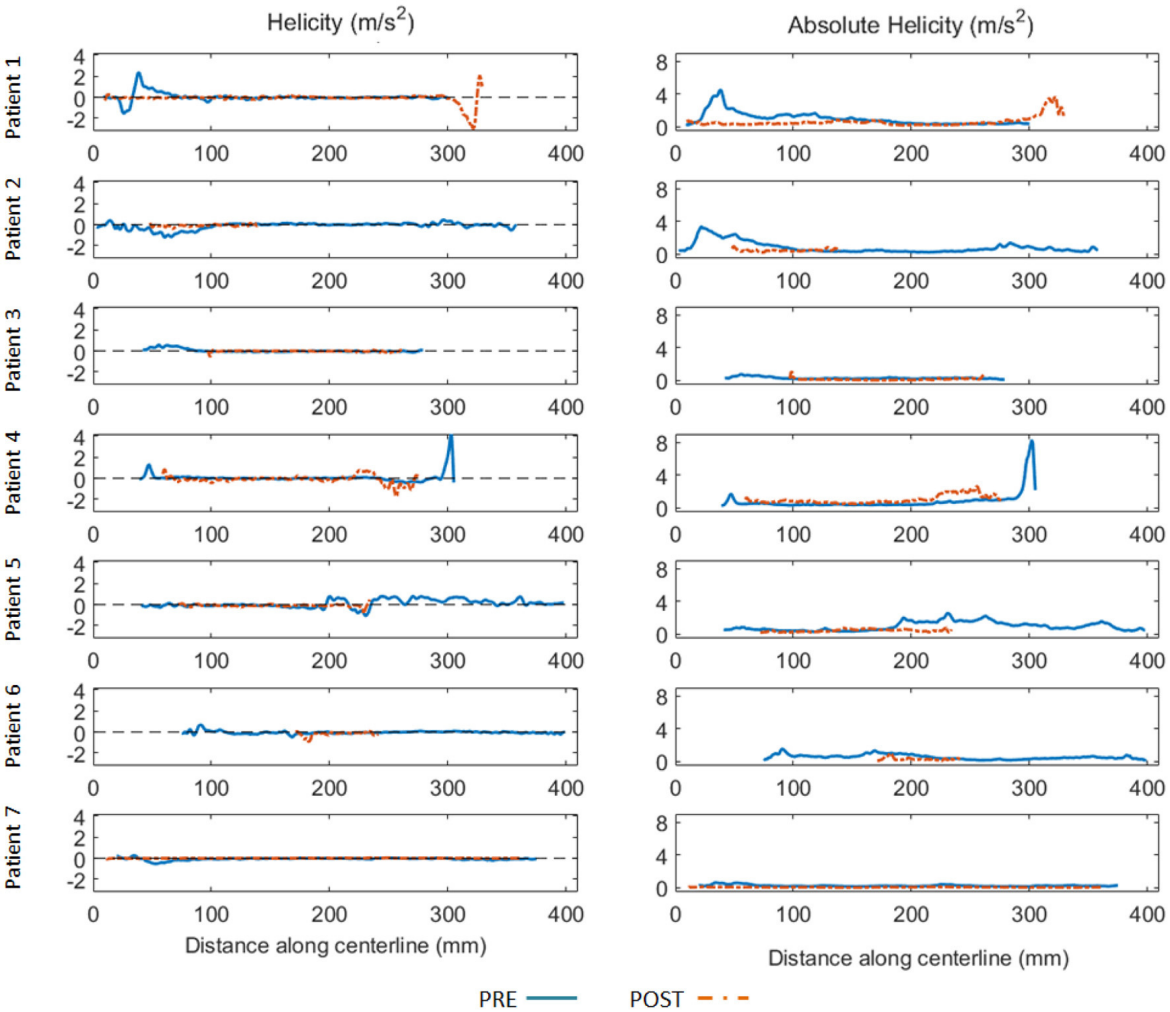
Helicity (left) and absolute helicity (right) plots obtained in the false lumen along the aorta centerline (spaced 1 mm apart) for all patients before (blue full line) and after TEVAR (orange dashed line). Horizontal axis indicates the distance (in mm) along the centerline with respect to the same anatomical reference location (5 cm above the aortic sinus). To be noted the elevated helicity in the proximal part of the false lumen for Patient 1 and Patient 2, indicating significant blood circulation through the entry tears. Elevated helicity was also observed in the distal part of the false lumen, related to one significant re-entry tear at the coeliac truncus (Patient 4) and to several entry tears in a patient with identified Marfan syndrome (Patient 5). Helicity decreased significantly post TEVAR in the proximal part of the false lumen for all patients. Oppositely, helicity increased in the distal part for 2 patients (Patient 1 and 4).

At qualitative inspection, helical flows were identified pre-operatively in 6 out of the 7 investigated patients using streamline visualization. Quantitatively, helicity was elevated in the proximal part of the false lumen in the majority of cases. The highest absolute helicity was observed in Patients 1 and 2, indicating significant blood circulation through the main entry tears. Elevated helicity was also observed in the distal part of the false lumen, related to one significant re-entry tear at the coeliac truncus (Patient 4) and to several entry tears in a patient with identified Marfan syndrome (Patient 5). Helicity decreased strongly post TEVAR in the proximal part of the false lumen for all patients. However, helicity increased in the distal part for Patients 1 and 4, which developed new post-operative entry tears (Figure 1). The results of the quantitative analysis of helicity are summarized in Figure 2.

Flow values obtained intra-stent (pulmonary artery level) were not significantly different than values in stent free regions (diaphragmatic level) with a median difference of 0.1 (IQR 0.3) L/min and an intraclass correlation coefficient equal to 0.967 (*p* < 0.01).

**Figure 3:**
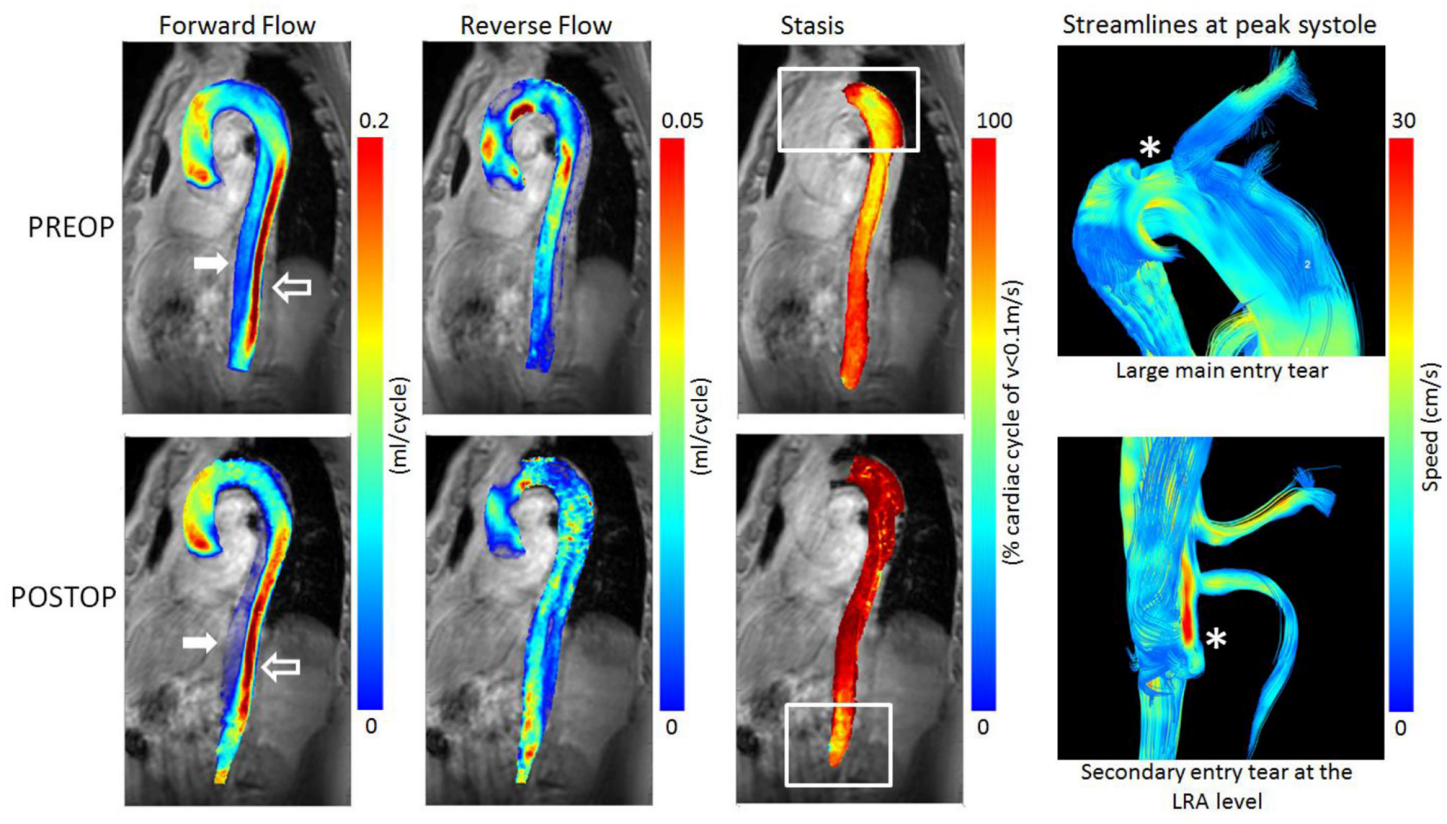
Pre-operative (TOP) and post-operative (BOTTOM) parametric hemodynamic maps of Patient 1 presenting with persistent pain as indication for TEVAR. Forward and Reverse flow maps (Left) are presented for both the true lumen (empty arrow) and the false lumen (white arrow). Forward flow increased in the true lumen and significantly decreased in the false lumen post-TEVAR. Similarly, reverse flow increased in the true lumen and decreased in the false. Reverse flow can also be observed in the ascending aorta indicating a recirculation region, both pre and post-op. To be noted, the local high reverse flow at the LRA level post-TEVAR that may indicate a hemodynamically significant secondary tear. Stasis maps (Center) are presented for the false lumen only to avoid confusion from the true lumen, as the false lumen twirls around it. Significant increase in stasis is observed post-TEVAR, except for the lower most region. Importantly, stasis maps facilitated visualization of hemodynamically significant entry tears (*), as confirmed by the streamline visualization (Right).

Typically, forward flow increased in the true lumen and significantly decreased in the false lumen post-operatively. Similarly, reverse flow increased in the true lumen and decreased in the false (Figure 3, patient presenting with malperfusion). Importantly, the stasis maps facilitated visualization of entry tears, as confirmed by the streamline visualization.

**Figure 4:**
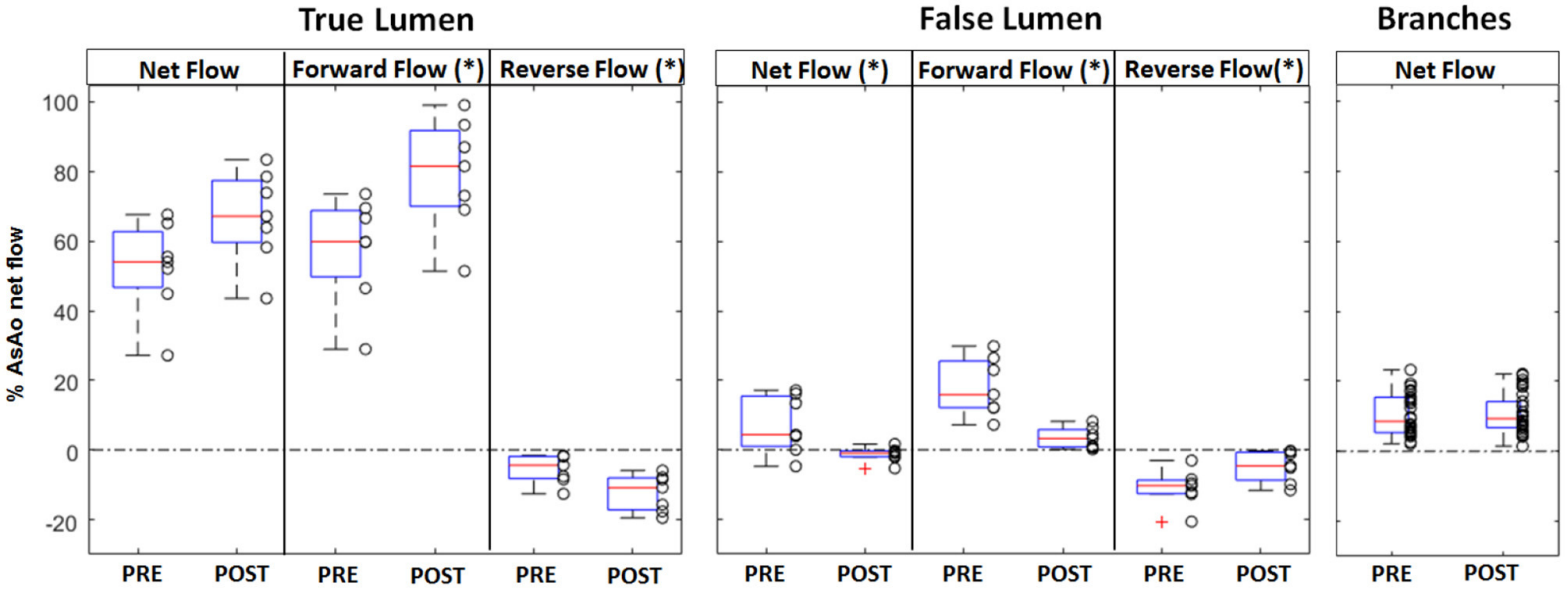
Box plot representation of the net, forward, and reverse flows in the true and the false lumen, and the net flow in the aortic branches, measured pre and post-intervention. The box plot shows the first and third quartiles, the red line shows the median, and the whiskers show the minimum and respectively maximum value; outliers are shown as red crosses. For the aorta, each value represents the average flow along the vessel centerline between the left subclavian artery and the celiac trunk and is normalized to the net flow of the ascending aorta. Significantly different flow values were obtained post-operatively in the false lumen for net, forward, and reverse flow, and in the true lumen for forward and reverse flow (* indicates *p* < 0.05).

In the true lumen, the net flow increased from 54.0% (IQR 16.0) to 67.2 % (IQR 17.8, *p* = 0.097) after endovascular treatment, the forward flow increased significantly from 59.9% (IQR 19.0) to 81.6% (IQR 21.8, *p* = 0.04), and the reverse flow increased significantly from 4.36% (IQR 6.4) to 10.8 % (IQR 9.2, *p* = 0.03). Oppositely, in the false lumen, the net flow decreased significantly from 4.4% (IQR 14.5) to 0.9% (IQR 1.7, *p* = 0.03) post-operatively, the forward flow decreased significantly from 15.9% (IQR 13.5) to 3.3 % (IQR 5.0, *p* < 0.01), and the reserve flow decreased significantly from 10.3% (IQR 3.8) to 4.6 % (IQR 8, *p* = 0.05) (Figure 4).

In the aortic branches, the changes in net flow were not significant (*p* > 0.05). The net median flow increased from 17.5 % (IQR 2) to 19.7% (IQR 6.8) in the truncus caeliacus (TC) post treatment, decreased from 9.8 % (IQR 9.6) to 7.7 % (IQR 5.9) in the superior mesenteric artery (SMA), and increased from 6.5 % (IQR 2.8) to 7.9 % (IQR 4.5) in the right renal artery (RRA) and from 5.7 % (IQR 1.3) to 6.1 % (IQR 4.7) in the left renal artery (LRA).

A relative median increase in the net flow of 16.1 % (IQR 54.4) was obtained for the true lumen and a median decrease of−112 % (IQR 33.3) for the false lumen. Similar to the true lumen, a relative median increase in the net flow of 8.3 % (IQR 38.5) of the TC, of 17.7 % (IQR 97.6) of the SMA, of 3.7 % (IQR 55) of the RRA, and of 15.9 % (IQR 60.6) of the LRA.

A significant increase in stasis of the false lumen (average of entire FL volume) was observed from 89 (IQR 15.2) pre-operatively to 95.1 (IQR 6.3) post-operatively (*p* = 0.04).

## Discussion

In the present study, we performed an exploratory analysis of the impact of endovascular treatment on the flows in the true and the false lumen, and aortic branches, in type B aortic dissection before and after treatment. We demonstrated that in the false lumen, endovascular treatment decreases the helicity, decreases significantly the flow, and increases the stasis. In true lumen, endovascular treatment increases the flow.

Type B aortic dissection patients have excessively high long-term morbidity and mortality rates (2–4, 6). This poor outcome is a consequence of the aneurismal evolution of the aorta that leads to re-interventions and even aortic ruptures (2, 3, 6). In current clinical practice, aneurysmal evolution is monitored with annual CTA or MRA. The parameters used to assess the risk of aneurysm evolution (patient's clinical history and the vascular morphology) (17, 18), do not provide quantitative hemodynamic information on the dissection, which is in essence a hemodynamic pathology. Yet, aneurysmal progression is likely a consequence of disturbed flow patterns. In this context, helical flow might be an important hemodynamic marker.

Investigation of aortic helical flow using 4D Flow, in healthy and disease cases, is generally based on the qualitative description of the flow pattern using vector, streamlines, and or pathlines visualization. The obtained information is the presence, overall magnitude, and direction of rotation of the helical flow (9). However, this visual analysis is user-dependent and challenging. Helical flow patterns can be missed during this visual inspection. In our patient population, we observed helical flow patterns in the proximal part of the false lumen in each dissection that had aneurysmal evolution as an indication for TEVAR (2 patients). These results are in agreement with Clough et al. (9) who showed a correlation between helical flow and aneurismal evolution. In that work, helicity was semi-quantitatively assessed as the amount of rotation over the cardiac cycle. In our study, we performed quantitative analysis of helicity and absolute helicity in the entire false lumen. We then presented the values per cross-section along the aorta centerline. Helicity quantification appeared helpful in detecting helical patterns, particularly at the level of secondary entry tears which had been missed by streamline visualization (Patient 2 and 5). Importantly, in our patient population, TEVAR resulted in the formation of new entry tears downstream of the stent in two patients (1 and 4), which were also missed at visual assessment of the streamlines. The formation of helical flow related to these new entry tears might suggest the patients would benefit from a closer follow up. However, given our small patient population, we cannot draw any conclusions on associations between the amount of helicity and the indications for TEVAR.

In addition to helicity, the global quantitative analysis also demonstrated complex flow patterns, particularly recirculation regions. The TEVAR procedure resulted in a significant increase in the forward flow in the true lumen, and a significant decrease of both forward and reverse flows in the false lumen. Interestingly, the reverse flow in the true lumen increased post TEVAR. This finding could be explained by the increase in the rigidity of the aorta wall, due to the presence of the metallic stent. Consequently, the aorta is no longer able to perform its function of dampening the pulse wave. Given the deleterious effects of vessel wall rigidity on patient outcome, particularly in the context of hypertension, we believe this finding should be investigated in a larger patient population, and its effect taken into account in the risk assessment.

Finally, it is important to note that the stasis maps enabled a straightforward detection of hemodynamically significant entry and re-entry tears and the related recirculation regions. We concluded to the presence of stasis when the flow was inferior to 0.1 mL/s during more than 90% of the cardiac cycle. Stasis can be interpreted as thrombus in process of creation, a criterion of success of endovascular treatment (12). Importantly, we noted that closure of the proximal entry tear resulted in an increase in flow through secondary re-entry tears when present downstream of the stent. This effect was easily identifiable both on the stasis maps and the absolute helicity maps.

Previous imaging based investigations of aortic dissection treatment were focused on the morphological information alone. Only one study presented quantitative flow measurements in the aorta before and after treatment, but this study was limited to only one patient and to relatively simple flow measurements. Importantly, flow measures performed intra-stent were not significantly different than measures in adjacent stent-free regions. This demonstrates that reliable velocity quantification was possible even in the presence of a metallic stent. Furthermore, our flow measurements in the aorta and its branches are in agreement with previous studies using 4D Flow in aortic dissections. In the aorta, at the level of the pulmonary artery, Clough and colleagues, found in 12 patients with chronic type B thoracic aortic dissection, a median stroke volume of 54.3 (IQR 43.2–64.8) mL/cycle in the true lumen and 31.6 (IQR 19.8–47.6) mL/cycle in the false lumen (9). Preoperatively, at the same level, we found a median stroke volume of 48.5 (IQR 17.8) mL/cycle in the true lumen and 4.5 (IQR 12.5) mL/cycle in the false lumen. In the TC and SMA, Siedek and colleagues found, in 22 healthy volunteers, a mean stroke volume of 12 +/- 5.3 mL/cycle (19). We found 14.95 (IQR 2.85) mL/cycle in the TC and 7.85 (IQR 6.35) mL/cycle in the SMA in our patient population preoperative. In the renal arteries, in a study on the repeatability and internal consistency of abdominal 2D and 4D Flow MRI, Wentland et al. (20) found in eight patients a mean stroke volume equal to 5.1 +/- 1.5 mL/cycle for the LRA and 5.9 +/-1.3 mL/cycle for the RRA. Similarly, we found a mean stroke volume of 5.6 (IQR 2.9) mL/cycle in the LRA and 4.4 (IQR 3) mL/cycle in the RRA.

Stability of the flow in the aortic branches is also an important outcome. Indeed, it reflects the correct perfusion of the branches and therefore the correct implantation of the endo-prosthesis. Furthermore, there is always a risk of malperfusion after occlusion of the false lumen when aortic branches are vascularized by this lumen. That is why it is important to ensure good vascularization of the aortic branches postoperatively.

Our study has some limitations. First, the size of the cohort is relatively small, with only eight patients included. This is because AD requiring endovascular treatment is not a very frequent condition, particularly at a single clinical center. Another limitation could be the heterogeneity of the cohort in terms of indication of endovascular treatment (cf Table 1): AD chronicity (2 acute, 6 chronic), entry tear (location, size, number), extent (type I, II or III DeBakey classification), and variability in intra-aortic flow. It should be noted that we chose to present our results in percentage of the ascending aorta net flow in order to avoid variability between patients (secondary to gender or body mass index for example) and to avoid variability between the two exams for the same patient. In addition, in our study we used a large FOV to cover the aorta down to the renal arteries. Measurements at the edge of the field-of-view, in the renal arteries for example, might suffer errors dues to gradient non-linearity. In addition, measurements in the renal arteries and the other aortic branches had lower VNR (velocity to noise ratios) than the aorta with the single-VENC (velocity encoding) acquisition that we used. A dual-VENC acquisition would have been ideal, however this strategy requires an almost double acquisition time compared to single-VENC, making it unfeasible in our setting. Furthermore, patient motion might have affected the measurements, since 4D Flow was a lengthy acquisition. Nevertheless, the flow rates we measured are in agreement with previously mentioned studies, demonstrating reliability of the 4D Flow acquisition. Another potential limitation may come from the manual segmentation of the time averaged PCA. While this is the standard approach for analyzing 4D Flow MRI, it may be difficult in regions of complex and low flow in the false lumen, thus segmentation errors cannot be excluded.

In conclusion, we demonstrated the hemodynamic impact of the endovascular intervention in type B aortic dissections using 4D Flow MRI. By providing an early qualitative and quantitative hemodynamic evaluation of the aorta and its branches, 4D Flow MRI may be helpful in the management of aortic dissection.

## Data Availability Statement

The original contributions presented in the study are included in the article, further inquiries can be directed to the corresponding author.

## Ethics Statement

The studies involving human participants were reviewed and approved by Hospices Civils de Lyon. The patients/participants provided their written informed consent to participate in this study.

## Author Contributions

BC, MS, LB, and PD contributed to conception and design of the study. BC acquired the data, organized the database, and performed data analysis. MS performed data and statistical analysis. ED, AM, and FF were significantly involved in the data analysis. BC and MS wrote the manuscript. All authors contributed to manuscript revision, read, and approved the submitted version.

## Conflict of Interest

The authors declare that the research was conducted in the absence of any commercial or financial relationships that could be construed as a potential conflict of interest.

## Publisher's Note

All claims expressed in this article are solely those of the authors and do not necessarily represent those of their affiliated organizations, or those of the publisher, the editors and the reviewers. Any product that may be evaluated in this article, or claim that may be made by its manufacturer, is not guaranteed or endorsed by the publisher.

## Abbreviations

MRI: magnetic resonance imaging
TBAD: type B aortic dissection
TEVAR: thoracic endovascular aortic repair
AD: aortic dissection
4D flow: 4D phase contrast magnetic resonance angiography
4D TRAK: dynamic gadolinium enhanced angiography
FLEF: false lumen ejection fraction
FF: forward flow
RF: reverse flow
TL: true lumen
FL: false lumen
TC: truncus caeliacus
SMA: superior mesenteric artery
RRA: right renal artery
LRA: left renal artery.
